# Hepatitis B virus infection and the risk of nonalcoholic fatty liver disease: a meta-analysis

**DOI:** 10.18632/oncotarget.22364

**Published:** 2017-11-03

**Authors:** Jianping Xiong, Haoaohai Zhang, Yaqin Wang, Anqiang Wang, Jin Bian, Hanchun Huang, Ying Zheng, Xinting Sang, Yiyao Xu, Xin Lu, Haitao Zhao

**Affiliations:** ^1^ Department of Liver Surgery, Peking Union Medical College Hospital, Chinese Academy of Medical Sciences and Peking Union Medical College, Beijing, China; ^2^ Department of Interventional Radiology, The First Affiliated Hospital of China Medical University, Shenyang, China; ^3^ State Key Laboratory of Quality Research in Chinese Medicine, Institute of Chinese Medical Science, University of Macau, Macau SAR, China

**Keywords:** hepatitis B Virus, nonalcoholic fatty liver disease, meta-analysis

## Abstract

Some studies have reported that hepatitis B virus (HBV) infection affects the risk of nonalcoholic fatty liver disease (NAFLD). However, this association is controversial. We conducted a systematic review and meta-analysis to investigate the relationship between HBV infection and NAFLD. Relevant studies published before May 2017 were identified by searching PubMed, EMBASE, and ISI Web of Science. We used the random-effects model proposed by DerSimonian and Laird to quantify the relationship between HBV infection and risk of NAFLD. We also conducted subgroup and sensitivity analyses to validate the stability of the results. Five articles, comprising 8,272 HBV-infected patients and 111,631 uninfected controls, were included in our research. Our meta-analysis suggested that the risk of NAFLD was significantly lower in HBV-infected patients than in uninfected controls, with heterogeneity between studies (summary odds ratio [OR] = 0.71; confidence interval [CI] = 0.53–0.90; I^2^ = 75.2%). However, the inverse relationship was observed in only cohort (OR = 0.83; 95% CI = 0.73–0.94) and cross-sectional studies (OR = 0.63; 95% CI = 0.47–0.79), not case-control studies (OR = 3.96; 95% CI = 2.10–7.48). In conclusion, HBV infection was inversely associated with the risk of NAFLD.

## INTRODUCTION

Nonalcoholic fatty liver disease (NAFLD) is the most common cause of chronic liver disease, affecting approximately 20–25% of U.S. adults [1–3]. In addition, NAFLD is an independent risk factor that is associated with an increased incidence of cirrhosis and primary liver cancer. Studies have reported that up to 9% of patients with NAFLD progress to liver fibrosis, cirrhosis and eventually hepatocellular carcinoma [4–7]. However, the pathogenesis of NAFLD is not well understood. Only a few risk factors have been recognized, including obesity, diabetes mellitus, and hyperlipidemia, and most of these factors are metabolically related [8–10]. Thus, an increasing number of researchers consider NAFLD to be a feature of metabolic syndrome [11–13]. Recent studies have reported an inverse relationship between hepatitis B virus (HBV) infection and metabolic syndrome [14, 15]. However, few studies have examined the effect of HBV infection on the risk of NAFLD, and the association between HBV infection and NAFLD remains controversial. HBV infection is a primary cause of the global health burden. An estimated 350 million people–i.e., 5%–7% of the world’s population–are chronic carriers of HBV, 75% of whom live in the Asia Pacific Region [16, 17]. Moreover, HBV infection is the leading cause of chronic liver disease, especially cirrhosis and hepatocellular carcinoma [18]. At least a third of patients with cirrhosis and 75% of patients with primary liver cancer have HBV [19, 20], and approximately 1 million people die of acute and chronic HBV infection every year [21]. Accordingly, we conducted a systematic review and meta-analysis of published observational studies to better understand the relationship between HBV infection and the risk of NAFLD.

## RESULTS

### Study selection and study characteristics

**Figure 1:**
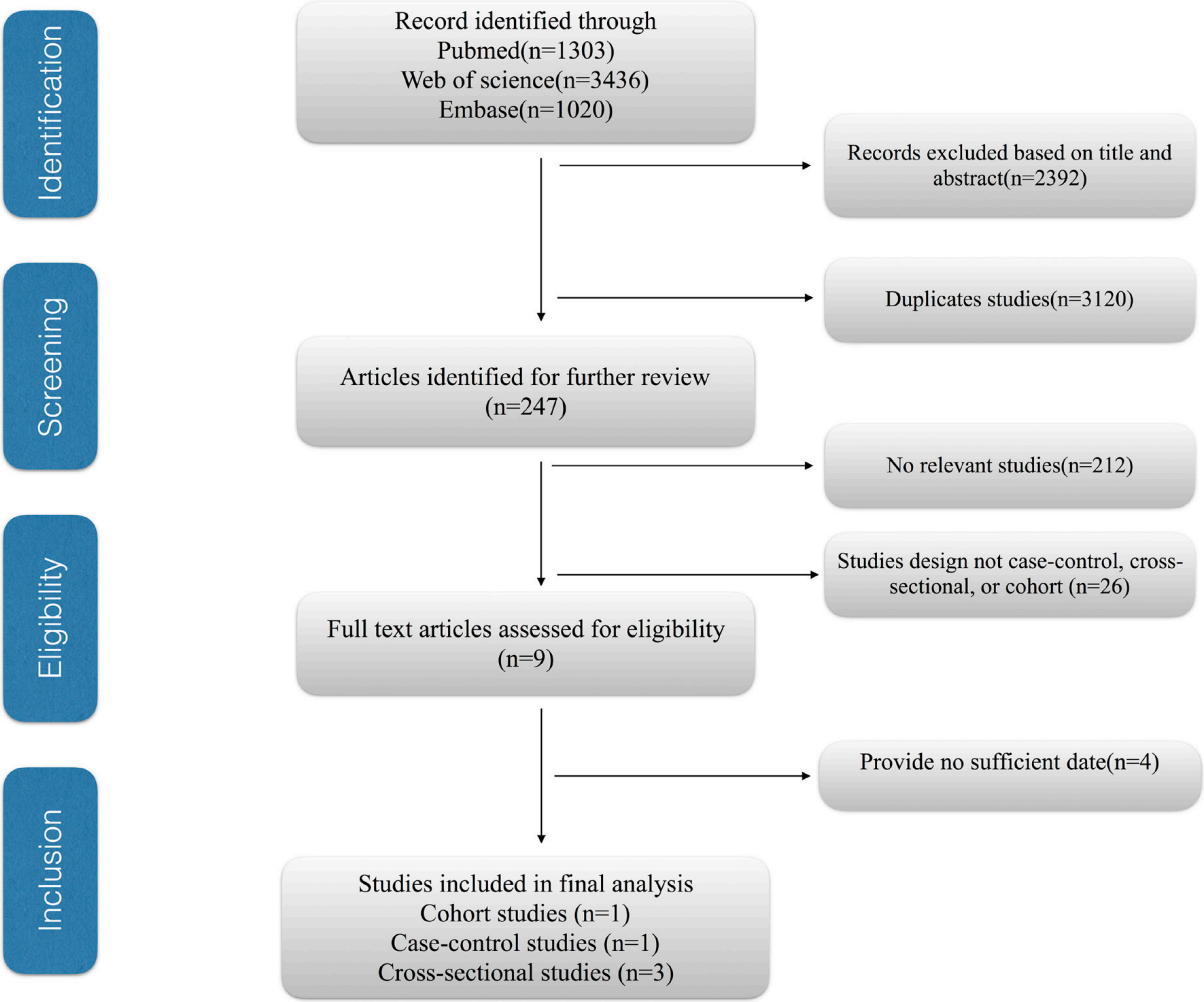
The study selection process for the meta-analysis

Figure 1 shows the study selection process. We obtained 5759 articles through the initial search (1303 from PubMed, 3436 from EMBASE, 1020 from Web of Science), 3120 of which were duplicates. We excluded 2392 more studies after reviewing the titles and abstracts, and three more studies were further excluded due to having insufficient information such as date unavailable, not relevant exposure and not relevant outcome [22–25]. Therefore, five eligible observational articles were included in our meta-analysis [26–30].

The main characteristics of the included studies are listed in Table 1 [26–30]. Two studies were performed in Taiwan, one in China, one in Korea and one in Hong Kong. All included studies were observational studies and included three cross-sectional [26, 27, 30], one case-control [29] and one cohort study [28]. The meta-analysis included 8,272 patients with HBV infection and 111,631 uninfected controls to explore the effect of HBV infection on the risk of NAFLD. The data analyzed in the studies were collected from 2002 to 2014. The modified NOS scores of the included studies ranged from 5 to 9, with four high-quality studies and only one of medium quality (Table 3).

**Table 1:** The main characteristics of the included studies

HBV and NAFLD									
Study/Years of Publication	Country	HBV+/HBV-	Follow	Sources of Controls	Outcome	Subtype of study	Exposure	Adjusted Factors	Adjusted OR/RR (95% CI)
Vincent Wai-Sun Wong.2012	Hong Kong	91/922	2008-2010	population	NAFLD	cross-sectional	HBV	alcohol consumption, demographic, and metabolic factors	0.42 (0.20, 0.88)
Yuan-Lung Cheng.2013	Taiwan	3642/29797	2002-2009	hospital	NAFLD	cross-sectional	HBV	age, sex, BMI,WC,SBP, fasting glucose, cholesterol, LDL, TG, ALT, GGT, platelet counts, FLI	0.66 (0.59, 0.72)
Eun-Jeong Joo.2016	Korea	3926/79413	2002-2014	population	NAFLD	cohort	HBV	age, sex, year of visit, smoking status, alcohol intake, regular exercise, education level, and body mass index	0.83 (0.73, 0.94)
Chia-Chi Wang.2007	Taiwan	50/457	NR	hospital	NAFLD	case-control	HBV	age, sex, ALT, riglyceride, total cholesterol, Body mass index, fasting blood glucose	0.97 (0.48, 1.95)
Peng XE.2013	China	527/1042	2007-2008	hospital	NAFLD	cross-sectional	HBV	NR	3.96 (2.10, 7.48)

RR, relative risk. OR, odds ratio. CI, confidence interval. NAFLD, nonalcoholic fatty liver disease. NR, no report. BMI, body mass index. WC, waist circumference. SBP, systolic blood pressure. HDL, high-density lipoprotein. LDL, low-density lipoprotein. TG, triglyceride. ALT, alanine aminotransferase. GGT, gamma-glutamyltransferase.

**Table 2:** Subgroup and sensitive analyses for HBV infection on the risk of NAFLD

Subgroup	No. of studies	RR (95%CI)	I^2^ value (%)	*P* value
All studies	5	0.71 (0.53, 0.90)	75.2	0.006
**Study quality**				
≥ 7	4	0.70 (0.55, 0.86)	71.3	0.015
< 7	1	3.96 (2.1, 7.48)	—	—
**Study design**				
Cohort	1	0.83 (0.73, 0.94)	—	—
Case control	1	3.96 (2.10, 7.48)	—	—
Cross sectional	3	0.63 (0.47, 0.79)	21.9	0.278
**Adjustment for confounders**				
**Alcohol intake**				
Yes	2	0.66 (0.26, 0.92)	80.4	0.024
No	3	1.02 (0.23, 1.81)	69.0	0.040
**Cholesterol level**				
Yes	3	0.63 (0.47, 0.79)	71.3	0.015
No	2	0.92 (0.81, 1.61)	80.7	0.203
**Diabetes**				
Yes	3	0.63 (0.47, 0.79)	21.9	0.278
No	2	0.92 (0.81, 1.61)	80.7	0.203
**Diagnosis of fatty liver**				
Ultrasound	4	0.77 (0.57, 0.97)	77.6	0.004
H-MRS	1	0.42 (0.20, 0.88)	—	—
**Sensitive analyses**				
High quality studies	4	0.70 (0.55, 0.86)	71.3	0.015
Excluding the study using MRS	4	0.77 (0.57, 0.97)	77.6	0.004
**Fixed-effects vs random-effects model method**				
Fixed-effects model	5	0.70 (0.65, 0.76)	75.2	0.003
Random-effects model	5	0.71 (0.53, 0.90)	75.2	0.003

HBV, hepatitis B virus. NAFLD, nonalcoholic fatty liver disease. RR, relative risk; CI, confidence interval.

**Table 3:** Scores of the modified Newcastle-Ottawa scale for studies

Study/Years of Publication	Fully defined cases	Define the study design	Selection of controls	Described the general characteristics	Controlling the important factors or confounding factors.	List inclusion and exclusion criteria for all the participants	Provided enrollment duration for all the participants	Indicate study period and follow-up duration	Total score
Vincent Wai-Sun.2012	^*^	^*^		^*^	^**^	^*^	^*^	^*^	8
Yuan-Lung Cheng.2013	^*^	^*^		^*^	^**^	^*^		^*^	7
Eun-Jeong Joo.2016	^*^	^*^	^*^	^*^	^**^	^*^	^*^	^*^	9
Chia-Chi Wang.2007	^*^	^*^		^*^	^**^	^*^	^*^		7
Peng XE.2013	^*^	^*^	^*^	^*^				^*^	6

The asterisks represent a score (number of stars).

### Association between HBV infection and risk of NAFLD

**Figure 2:**
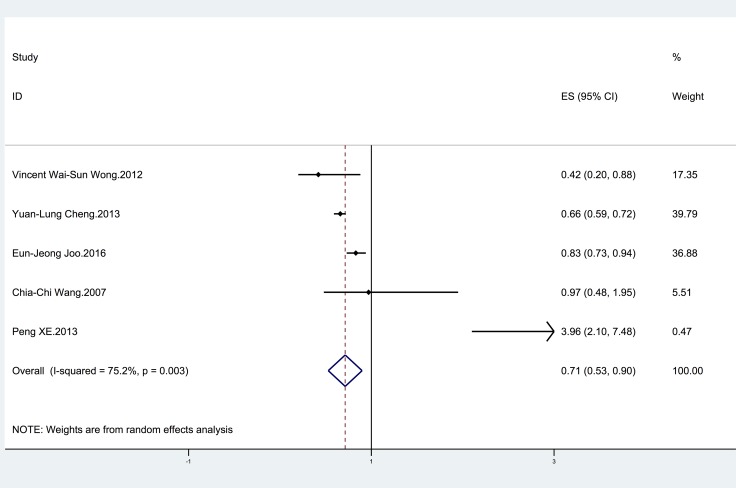
Forest plot showing the relationship between HBV infection and the risk of NAFLD The horizontal axis is the X axis. The points represent the risk estimate of each individual study. Horizontal lines represent 95% confidence intervals; diamonds represent the summary risk estimate with 95% confidence interval. HBV, hepatitis B virus. NAFLD, nonalcoholic fatty liver disease. CI, confidence interval. ES, effect size.

Five studies [26–30] were included to investigate the relationship between HBV infection and the risk of NAFLD (Table 1). Three studies [26–28] suggested that HBV infection was associated with a decreased risk of NAFLD. Only one study [30] reported a significantly higher risk of NAFLD in HBV-infected patients than in uninfected controls. The remaining studies [29] did not show a significant relationship. The pooled estimate was significant (OR = 0.71; 95% CI = 0.53–0.90 ), with significant heterogeneity (I^2^ = 75.2%; *p* = 0.003) (Figure 2).

### Subgroup and sensitivity analyses

The results of the subgroup analyses and sensitivity analyses are shown in Table 2. When the analysis was stratified by study quality, study design and adjustment for cholesterol level or diabetes in the models, there was a significant difference between subgroups (*P* < 0.05) (Table 2). For example, HBV infection was significantly associated with the risk of NAFLD in cohort (OR = 0.83; 95% CI = 0.73–0.94) and cross-sectional studies (OR = 0.63; 95% CI = 0.47–0.79), but not in case-control studies (OR = 3.96; 95% CI = 2.10–7.48) (Table 2). According to the sensitivity analyses, despite excluded the study using MRS, the results of the relationship between HBV infection and NAFLD remained stable (OR = 0.77; 95% CI = 0.57–0.97). Additionally, the overall results remained consistent when the pooling model was changed (fixed-effects model: OR = 0.70; 95% CI = 0.65–0.76 and random-effects model: OR = 0.71; 95% CI = 0.53–0.90).

### Publication bias

The number of studies included in this meta-analysis was insufficient to assess publication bias (< 10).

## DISCUSSION

To our knowledge, this is the first meta-analysis to investigate the relationship between HBV infection and the risk of NAFLD. Five studies were included to examine the effect of HBV infection on the risk of NAFLD, and the analyses showed that the risk of NAFLD was significantly lower in the 8,272 HBV-infected patients than in the 111,631 uninfected controls (OR = 0.71; 95% CI = 0.53–0.90), with significant heterogeneity between studies. This effect was observed in cohort and cross-sectional studies, but case-control studies did not reveal an inverse relationship between HBV infection and the risk of NAFLD.

Our study demonstrated only an association between HBV infection and a reduced risk of NAFLD; the data could not establish a causative role of HBV in this regard. The potential biological mechanisms of this finding include the following. Studies have reported that patients with HBV infection have a lower level of triglycerides, which may affect the development of NAFLD [31]. Additionally, studies have suggested that HBV X protein can inhibit the secretion of apolipoprotein B, which is an important component of the formation of very-low-density lipoprotein and low-density lipoprotein [32, 33]. In addition, other studies have indicated the association between HBV seropositivity and low serum cholesterol levels [34, 35].

Our study has several strengths. First, it is the first meta-analysis with a large sample size (8,272 HBV-infected patients and 111,631 uninfected controls) to evaluate the effect of HBV infection on the risk of NAFLD. Therefore, the findings may provide insight into the relationship between HBV infection and NAFLD. In human studies, HBV infection affects the secretion of various adipokines and may alter the lipid profile [36, 37]. As an analogy, lipid metabolism has been implicated in hepatitis C viral entry, replication and response to treatment [38]. Hepatitis B virus replication may affect lipid metabolism and this warrants further studies. From a clinical standpoint, further studies on the mechanism linking lipid metabolism and HBV replicative cycle may shed light on new treatment targets on NAFLD [26]. Second, subgroup and sensitivity analyses were performed to determine the factors that may have affected the results and improved the reliability of our findings. Third, we performed a comprehensive literature search of the PubMed, EMBASE and Web of Science databases to identify potential studies to investigate the relationships between HBV infection and the risk of NAFLD. In addition, most of the studies included in our meta-analysis were of high quality. These characteristics make the conclusions of our study more persuasive.

There are several limitations that should be considered. First, the studies included in our meta-analysis used different methods to diagnose the NAFLD outcomes, including ultrasound and proton-magnetic resonance spectroscopy (H-MRS) [39]. The results of studies with different diagnostic measures were combined, which leads to concern regarding heterogeneity in the meta-analysis. In addition, the methodological differences may limit the comparability of studies and influence the impact identified on NAFLD risk. Second, the findings presented only an association, which is subject to confounding bias. Although we considered and discussed a number of adjustment factors, many potential adjustment factors were not included, such as physical activity and other dietary factors. For example, hepatitis B patients may pay more attention to physical activity and have good dietary habits, which have been shown to affect the development of NAFLD in previous studies [40–43]. In addition, we failed to obtain information about HBV-infected patients’ use of antiviral treatment, which may have influenced the development of NAFLD. Third, all of the included studies in our meta-analysis were performed in Asia, and it is thus difficult to generalize our findings to the general population. Finally, only five studies were included in our article, placing the meta-analysis at a high risk of publication bias. Additionally, due to the different study designs and demographic characteristics, the heterogeneity between studies was significant and may be considered another potential limitation of this study. In addition, most of the studies included in our meta-analysis were case-control and cross-sectional studies; case-control designs are prone to recall and selection biases and cross-sectional designs have insufficient power to evaluate the relationship between HBV infection and the risk of NAFLD.

In summary, our meta-analysis indicated that HBV infection is associated with a lower prevalence of NAFLD, and the inverse relationship was demonstrated in cohort and cross-sectional studies only, not in case-control studies. More prospective studies and basic research should be conducted to further validate the association between HBV and NAFLD and to examine the potential mechanisms involved.

## MATERIALS AND METHODS

### Data sources and search strategy

We searched published reports in the PubMed, EMBASE and Web of Science databases using the following keywords: (“hepatitis B” OR “hepatitis B virus” OR “HBV”) and (“fatty liver” OR “nonalcoholic fatty liver disease” OR “NAFLD” OR “nonalcoholic steatohepatitis” OR “NASH”). We placed no restrictions on the language or date of publication.

### Eligibility criteria for study selection

The eligibility criteria were as follows: a cross-sectional, case control or cohort study design; HBV as the exposure factor and NAFLD as the outcome; and odds ratio (OR)/risk ratio (RR) values and corresponding 95% confidence intervals (CIs) in the HBV-positive and HBV-negative groups described or sufficient information to calculate them. If two studies reported the same data, we selected the study with the larger sample.

### Data abstraction and quality assessment

Two researchers independently extracted the required information from the selected studies in a standardized manner. We collected the following information from each article: first author’s name, year of publication, country of origin, study design (cross-sectional, case-control or cohort), number of participants, duration of follow-up, sources of controls, adjustment for confounding variables, and OR/RR values and 95% CIs in the HBV-positive and HBV-negative groups. HBV infection is defined as HBsAg positive.

A universal scale that assesses the quality of all types of observational studies is not available. Therefore, two authors independently used the modified Newcastle–Ottawa Scales (NOS) [44] reported by Wei Zhu [45] to evaluate the quality of the included studies. We assigned quality categories according to the scores of each study. Specifically, NOS scores of < 4, 4–6, and 7–9 indicated low-, medium-, and high-quality studies, respectively [46]. The maximum total score was 9 points. We resolved discrepancies by consensus.

### Statistical analyses

The OR/RR values and corresponding 95% CIs were used to evaluate the relationship between HBV infection and the risk of NAFLD. We treated hazard ratios as equivalent to RRs. We used the random-effects model proposed by DerSimonian and Laird to quantify the relationship between HBV infection and risk of NAFLD [47].

The I^2^ statistic was used to quantify the heterogeneity between studies, and I^2^ values of 25%, 50%, and 75% represented low, medium, and high heterogeneity, respectively [48]. *P* values less than 0.1 indicated that clear heterogeneity existed. As this meta-analysis included less than ten studies, a funnel plot was not performed to evaluate publication bias [49].

We also performed subgroup analyses by study quality, study design and adjustment for alcohol intake, cholesterol level, diagnosis of fatty liver or diabetes in the models. Sensitivity analyses were also conducted by changing the pooling model (random-effects model or fixed-effects model), excluding the study using MRS and excluding studies with NOS scores < 7.

All statistical analyses were performed using STATA version 12.0 (Stata).

## Abbreviations

HBV: hepatitis B virus
NAFLD: nonalcoholic fatty liver disease
